# Evolution of *Escherichia coli* Expression System in Producing Antibody Recombinant Fragments

**DOI:** 10.3390/ijms21176324

**Published:** 2020-08-31

**Authors:** Annamaria Sandomenico, Jwala P. Sivaccumar, Menotti Ruvo

**Affiliations:** Istituto di Biostrutture e Bioimmagini, CNR, via Mezzocannone, 16, 80134 Napoli, Italy; jwala.priyadarsini@gmail.com

**Keywords:** antibody fragment, Fab, scFv, *E. coli*

## Abstract

Antibodies and antibody-derived molecules are continuously developed as both therapeutic agents and key reagents for advanced diagnostic investigations. Their application in these fields has indeed greatly expanded the demand of these molecules and the need for their production in high yield and purity. While full-length antibodies require mammalian expression systems due to the occurrence of functionally and structurally important glycosylations, most antibody fragments and antibody-like molecules are non-glycosylated and can be more conveniently prepared in *E. coli*-based expression platforms. We propose here an updated survey of the most effective and appropriate methods of preparation of antibody fragments that exploit *E. coli* as an expression background and review the pros and cons of the different platforms available today. Around 250 references accompany and complete the review together with some lists of the most important new antibody-like molecules that are on the market or are being developed as new biotherapeutics or diagnostic agents.

## 1. Introduction

Antibody fragments are widely utilized in therapeutic and diagnostic applications as well as in basic life science research [1]. Unlike conventional immunoglobulins, these smaller biomolecules take several pharmacokinetics advantages over whole antibodies including better penetration into tissues, faster clearance for imaging purposes and generally lower immunogenicity. On the other hand, the absence of the Fc domain and the small size results in a shorter half-life compared to full-length antibodies [2].

In accordance with the antibody’s structure–activity relationship, the prerequisite for generating active and smaller antibody-mimicking molecules is the presence of the “antigen-binding site”, the tridimensional pocket arising from the variable domains of heavy (VH) and light (VL) chains. The target specificity is mediated by three peptide loops at the tip of each V-domain, designated as complementarity determining region (CDR). Together, these six CDR loops form the target-binding paratope or idiotype of an antibody. For proper target binding, the two V-domains need to pair up in the proper orientation so that the CDR loops jointly form a specific paratope.

Despite that several alternative antibody-like formats derived from different Ig-like domain combinations are continuously developed and proposed [1] such as single-domain antibody fragments (dAbs), the antigen-binding fragments (Fab) and single-chain variable fragments (scFv) are those most used and widespread.

Owing to the lack of glycosylation and their size, these small antibody-like molecules can be readily produced in active and functional recombinant forms via *E. coli* prokaryotic expression systems which enable easier production and with low costs compared to other available expression platforms like yeasts, insect cell lines, mammalian cells and transgenic plants and animals [3].

Single-domain antibody fragments (dAbs), also known as nanobodies, consist of VH or VL domains of 12–15 kDa and are the smallest functional antibody fragments that retain full antigen-binding specificity. Given their high-affinity, solubility and stability also in absence of the partner VL domain, the camelid VH domains (VHH) [4] and the shark VH domains called V-NAR (New Antigen Receptor) [5], are currently used as templates to generate highly efficient and stable new antibody fragments collectively termed nanobodies [6,7,8]. The natural evolution of VHH and V-NAR Ig domains, derived from the respective HCAbs (heavy-chain Abs that lack light chains), is to deliver new structural solutions for overcoming limitations such as Ig folding stability and antigen affinity. This can be achieved through the design of recombinant “super stable” Ab-fragments, optimizing the hydrophobic VH/VL interfaces and the CDR loops [9,10]. Compared to the VH domains, the VHH and V-NAR domains contain more hydrophilic residues on the surface that interfaces with the VL domains, and some hydrophobic residues, that are fixed in the sequences, are analogous to those at the VH/VL interface and are used for interacting with hydrophobic residues in the CDR3 loop [11,12]. Moreover, the high-affinity binding of VHH and V-NAR domains is likely supported by an increased length of the majority of the CDR3 loops (3–28 amino acids) compared to that of the VH domains [13]. The extended CDR3 loop of nanobodies has the capacity to form a finger-like structure with greater structural flexibility which, in turn, is likely fixed in one single conformation upon antigen-binding [14,15].

The single-chain fragment variable (scFv) domains consist of a single polypeptide (25 kDa) in which the variable regions of the light (VL) and heavy chains (VH) are joined by a flexible linker resistant to endopeptidases. The sequence and length of the ideal linkers may differ between scFvs in order to optimize the affinity for the antigen, reduce the oligomerization and increase the thermostability [16]. It has been largely demonstrated that the linker length influences the oligomeric state. Linkers greater than 15 residues generally lead to monomers while linkers of 6–15 residues can be utilized to deliberately favor the formation of stable dimers and trimers [17,18]. Linkers with fewer than five residues result in the generation of higher-order multimeric molecules [19,20]. The length and amino acid sequence of the peptide linker are, therefore, crucial for proper domain orientation and for regulating their intramolecular or intermolecular interactions. In the presence of short linkers, one antibody fragment’s VL or VH domain interacts with another molecule’s complimentary domain through a mechanism known as “domain swapping” generating dimers or higher-order oligomers [21]. De facto, short or even medium linkers (up to 15 residues) hamper the proper rotation and intramolecular alignment of the covalently linked complimentary Ig domains preventing the formation of the correct interface. This configuration promotes a swap of the Ig domains leading to the intermolecular association between two, three or even four molecules of scFvs and the generation of the so-called diabodies, triabodies or tetrabodies, respectively. An interesting application of the Ig domain swapping in scFv molecules is the development of bispecific diabodies, especially as antitumor biotherapeutics [22,23]. One such diabody is Blinatumomab, a bispecific T-cell engager (BiTEs) which combines one binding site against CD3, occurring on T cells, and a second anti-CD19 site, specific for cancerous B cells. Blinatumomab is an FDA-approved drug used for treating B-ALL (B cell precursor acute lymphoblastic leukemia, ALL). In cancer immunotherapy, the scFv technology has been also adopted for the development of the CAR T cells (chimeric antigen receptor) technology. Both these strategies use scFvs to recruit cytotoxic T lymphocytes (CTL) in proximity of target tumoral cells that express specific surface antigens (i.e., CD19) and facilitate the polyclonal T-cell response to tumor antigens [24]. The most common linkers used for the generation of scFvs are glycine- and serine-rich amino acidic stretches having sequences (GGGGS)_3_ of 15 aa, or (GGGGS)_4_, of 20 aa. These linkers are widely used to keep the carboxy terminus of one variable domain and the amino terminus of the other at a distance that favors the correct folding and the formation of the antigen-binding site while minimizing, at the same time, the oligomerization [25,26]. Some reports have performed the screening of linkers in terms of length and amino acid composition in order to optimize the scFv fragments solubility and activity [27,28,29]. The order in which the heavy and light domains are fused to build the scFv varies throughout the literature. In some cases, VL-linker-VH [30] rather than VH-linker-VL shows favorable biophysical characteristics, while in other cases, the reverse is true [31]. In some cases, the two different formats exhibited the same antigen-binding activities [32].

Despite the relatively simple structure of scFv fragments, their practical use is limited by the aggregation propensity and consequent low homogeneity. This is due to their dynamic structural features (open and closed state) depending on interchain VH-VL interactions. To suppress this dynamic equilibrium the introduction of a disulfide bond at the VH-VL interface has been attempted. It has been found that the replacement of residues vH44 and vL100 leads to one of the most favorable disulfide configurations [33]. In this instance, the phage display method has been used to generate stable mutants [29,34]. Recently, the use of cyclic scFvs variants has been also successfully reported as an innovative strategy to suppress their intrinsic oligomerization tendency and to optimize the production of stable and active products in bacterial cytoplasm [35,36].

The Fab fragment is a heterodimeric and monovalent antibody fragment (50 kDa) composed of an antibody light chain (VL + CL domains) linked by a disulfide bond to the antibody heavy chain (VH + CH1 domains). Usually, Fab fragments are biochemically more stable than scFv counterparts due to the mutual stabilization that occurs between the VH/VL and CH1/CL interfaces [37]. In addition to the possible aggregation and degradation issues, also the production of Fab fragments in *E. coli* hosts results challenging. It is indeed necessary to achieve the “optimum” expression rather than the “maximum” expression of both chains because the best ratio of HC and LC and protein folding rates are needed and the separately expressed light and heavy chains must assemble correctly to constitute the functional heterodimer with four intrachain and one interchain disulfide bond [38].

In this field, significant efforts have been made to obtain satisfactory expression levels and to identify optimal processing and folding procedures. A comprehensive and progressive better understanding of the mechanisms by which the Ig domains fold and retain their native/functional conformation and of the stability and solubility in “host-environmental conditions” is however still required to improve the production and quality of antibody-based biomolecules. This is the first step towards large-scale productions for clinical applications. In the last decade, several tools including innovative cloning, expression and purification strategies have been explored to increase the production of functional antibody-like fragments using *E. coli* microbial platforms. Due to the ability of *E. coli* to grow at high cell densities, these biomolecules can be produced in high cell density cultures grown in stirred tank reactors using fed-batch methods. Moreover, in the case of scFvs and nanobodies, the reduced size and the single polypeptide nature make antibody fragments readily amenable to high-throughput selection technologies such as phage display, cell display, yeast display and ribosomal display [39]. Finally, these biomolecules obtained as recombinant proteins can be ad hoc engineered and specifically tuned to optimize serum half-lives, tumor penetration and clearance features by controlling their size through chemical or genetic modifications. These technologies include: (i) PEGylation, used for example to obtain certolizumab pegol, or Cimzia®, a marketed PEGylated anti-TNFα Fab for rheumatoid arthritis [40]; (ii) conjugation to the Fc domain of conventional antibodies; (iii) coupling to highly abundant and safe serum proteins such as human serum albumin (HSA), apolipoprotein L1 and β-Lactamase [41]; (iv) site-specific tagging for the generation of drug conjugated antibody fragments that promote tumor homing [42]; (v) generation of multifunctional and multispecific bigger molecules such as diabody (60 kDa), triabodies (90 kDa), tetrabodies (120 kDa), Fab dimers (100 kDa) and Fab trimers F(ab’)3 (150 kDa) [1].

The aim of this review is to revisit the current protein expression approaches using *E. coli* as host for the production of recombinant antibody-like fragments, especially Fab and scFv, and to address the development of new strategies based on both cell-based and cell-free systems. A brief survey of antibody fragments produced in *E. coli*, which are FDA-approved or are in the clinical phases, is also provided.

## 2. *E. coli* as Microbial Expression Stem for the Production of Antibody Fragments

*E. coli* is one of the most well-established cell factories for the production of recombinant proteins (RPP) [43]. Currently, many molecular tools and protocols are available for the high-level production of heterologous proteins, including a vast catalog of expression plasmids and of engineered strains and many cultivation strategies. From a theoretical point of view, the steps needed for obtaining a recombinant protein are pretty straightforward. The gene of interest (GOI) is cloned in whatever available expression vector, is transformed into the host of choice, expression is induced and the protein is then ready for purification and biochemical, structural and functional characterization. Practically, however, many things can go wrong such as poor growth of the host, inclusion body (IB) formation, protein inactivity, and even lack of protein expression. Choosing the perfect combination is not possible *a priori*, thereby multiple conditions should be empirically tested to obtain a soluble and active recombinant protein.

### 2.1. Features, Advantages and Disadvantages of the E. coli Microbial Expression System

Given the fast growth rate (doubling time is 20 min), the low cost (the medium is inexpensive), the easiness of genetic manipulation, the well-known molecular features, the good productivity and the simple fermentation process development for manufacturing scale-up, this microorganism represents an affordable expression system for recombinant protein [43,44,45,46]. In shake flasks, *E. coli* generally produces a low amount of proteins (mg/L). However, in fermenters, several grams in a liter (g/L) can be achieved [47]. Besides the above-mentioned benefits, there are some main and general drawbacks that can arise, like the lack of proper post-translational modifications (PTMs), the formation of inclusion bodies (IB), codon bias, metabolic burden (acetate accumulation) [48] and occurrence of proteins degradation.

In the last decades, numerous *E. coli* strains engineered to improve their efficiency in the production of recombinant proteins have been developed [49,50,51,52,53] and have been also largely used for the preparation of recombinant antibody-like fragments [54,55,56]. Some of them have also become the gold standard for biopharmaceutical applications while others have remained only tools for basic research [44]. Of interest, the “bacterial glycoengineering” is emerging as an advanced biotechnological approach that harnesses prokaryotic glycosylation systems for the generation of recombinantly glycosylated proteins using *E. coli* host [57]. In a recent exhaustive review, Harding and coworkers have described the oligosaccharyltransferase (OTase)-dependent (periplasmic) and OTase-independent (cytoplasmic) pathways which are recombinantly introduced into *E. coli* to produce N- or O-glycosylated recombinant proteins, including glycoconjugated vaccines and therapeutic proteins. The same approach can be potentially applied for N-glycosylation of monoclonal antibodies and antibody-related fragments [58,59].

Recently, Kulmala and coworkers have demonstrated that the “harmonized versions” of Fab fragments rather than the classic “over-optimized” one increases the expression from negligible levels to 10 mg/L [60]. Following the “codon harmonization” method [61], they have redesigned codon-optimized synthetic human Fab genes by making synonymous codon substitutions to only five segments of the Fab gene framework. Furthermore, they have also explored the effect of synonymous codon mutation of the pelB leader peptide in Fab periplasmic expression by a combinatorial approach [62].

The Progen company has instead developed the new expression vector pOPE101 [63] which is employed for the production of soluble and functional scFvs as well as for the generation of small fragment antibody libraries in *E. coli* [64]. The cassette vector pOPE101 contains a strong IPTG-inducible synthetic promoter, a pelB leader and a c-myc/His tag sequence for the secretion of functional recombinant proteins into the periplasmic space and to facilitate their detection and purification. The VH and VL genes are joined by a DNA-fragment coding for a flexible 18 amino acid linker containing the first six amino acids of the CH1 constant region domain and the hydrophilic pig brain alpha-tubulin peptide sequence EEGEFSEAR. This linker represents a valuable alternative amino acid sequence with respect to the classic Gly-Ser motifs used to get active and soluble scFv fragments [64].

Other reliable and controllable systems for positively regulating the expression of recombinant proteins in bacteria are based on l-arabinose or l-rhamnose operons. These systems are characterized by a slow response with very low basal transcriptional activity, which can be a great advantage for the production of proteins that are detrimental to the host cell.

In the pBAD vector systems [65], the GOI is placed downstream of the araBAD promoter. Its expression is activated in response to stimulation with l-arabinose and is inhibited by d-glucose that suppresses the basal expression due to a reduction in cellular cAMP levels. Similarly to the pBAD system, the l-rhamnose-inducible promoter pRha has also been successfully used for developing the Expresso® Rhamnose expression system to obtain high-level recombinant protein expression in the presence of l-rhamnose [66].

Protein expression using pBAD and pRha vectors is more tightly controlled compared to other expression systems such as pET vectors. The precise control of expression levels, based on catabolite repression, makes these systems ideal for producing problematic proteins, such as proteins with toxicity or insolubility issues. As a consequence of more stringent regulation of target gene expression, the attainable yields are relatively lower compared to those reached using pET systems. Through the literature, several attempts have been carried out to improve the production and yield of recombinant antibody fragments using these two vectors. Signal peptide sequences for these vectors have been also optimized following a stress minimization approach [67,68]. Karyolaimos and coworkers have obtained a yield of around 0.2 g/L of a functional scFv fragment in the periplasm using the OmpA signal peptide and 100 μM rhamnose as inductor [68].

Additionally, the Lemo system ™ [20], where the expression is under the tight control of the T7 RNAP activity and the target gene expression level is modulated by l-rhamnose, has been used for the production of soluble and properly folded scFv fragments [69,70].

For the first time, Petrus and coworkers have reported a robust scalable expression system for the production of scFvs in the periplasmic space based on the use of the innovative pSAR-2 vector. The pSAR-2 is an ad hoc engineered expression vector containing a rhamnose-inducible promoter (prhaBAD) and an N-terminal pelB signal peptide. The HIV-neutralizing PGT135 scFv antibody fragment was obtained with an outstanding yield of 1.2 g/L after 48 h of induction at 25 °C using 15 mM l-rhamnose in shake flasks [71].

Ten years ago, a novel tightly regulated expression system based on the chemical inducer cumate (4-isopropylbenzoate) was developed for high protein production in *E. coli* [72]. The corresponding pNEW vector contains in the expression cassette the regulatory elements of the Pseudomonas putida F1 cym and cmt operons. These two operons control the expression of the target gene at the transcriptional level by means of cumate. The constitutive expression of the desired gene is achieved through switching of the cumate-regulated gene that contains a partial T5-phage promoter merged with a synthetic operator and the repressor protein cymR. In the presence of cumate, the pNEW vector is able to increase the production yield of recombinant proteins by two to three-fold compared to pET-based IPTG-inducible systems. No specific examples of antibody fragment production have been so far reported; however, on the basis of the high-expression yields of the target protein, the tight regulation, the rheostatic control, and the homogenous high-expression bacterial culture, this vector promises to be the basis for innovative strategies to improve the production of antibody-like fragments.

In general, the plasmid-based expression systems exhibit some drawbacks, including the continuous amplification of the plasmid copy number in prolonged cultivations and loss of plasmids over time, propagating the generation of a plasmid-free subpopulation during induction [73]. In this context, the combination of plasmid-free [74] and cell-free approaches [71,75,76] represent new potential strategies for optimizing *E. coli* expression platforms (see the next section: New Platforms and new technologies for *E. coli*-based cell-free expression systems).

### 2.2. Inclusion Bodies

*E. coli* loses the spatiotemporal control of its own protein synthesis machinery when an exogenous gene is introduced. On the other hand, the newly synthesized recombinant polypeptide is expressed in the microenvironment of the host that may differ from that of the original source in terms of pH, osmolarity, redox potential, cofactors, and folding mechanisms. High local concentrations of the nascent heterologous protein together with an insufficient amount of folding-promoting chaperons may lead to partially folded or misfolded protein intermediates that give rise to the formation, in both the cytoplasm and periplasm, of insoluble protein aggregates known as inclusion bodies, IBs [77]. Usually, these intermediates expose on their surface hydrophobic patches that interact with similar regions and together with the formation of uncorrected disulfide bonds can lead to protein aggregation and precipitation Protein recovery strategies from inclusion bodies via solubilization and refolding processes are laborious, time-consuming and expensive, although various refolding procedures have been developed for therapeutic proteins and applied for antibody fragments [78,79,80]. An introduction to the IB refolding procedure [81] is outside the scope of this review. Rather, we will look at other more profitable and convenient strategies such as periplasmic and extracellular expression strategies and also co-expression with molecular chaperons for improving solubility and proper folding of antibody-like fragments, even on a laboratory scale.

### 2.3. Overcoming Drawbacks in Small-Scale Productions

After the transformation of the gene coding for the desired protein in the selected *E. coli* strain, a process development starts through small-scale cultures for the screening of expression conditions using plates and shake flasks. Various cultivation parameters, such as media composition, pH, agitation, aeration, temperature, cell density, concentration of inducers, induction time and strategies affect the protein expression level depending upon expression system [82]. The widely used standard procedures for shake flask cultures are described in the molecular cloning laboratory manual (“Sambrook protocol”) [83]. The most popular and suitable media for growing *E. coli* are Luria-Bertani (LB), Terrific Broth (TB) and Super Broth (SB) media that are easily prepared through different combinations of yeast extract, peptones and essential growth factors and vitamins. In the Studier’s autoinduction medium [84], the growth is supported at the beginning by glucose, and when glucose is exhausted protein expression is autoinduced by a diauxic shift to lactose utilization, while glycerol is also coutilized as a major carbon source during expression. The most recent EnBase^®^ medium (EnPresso, GmbH, Berlin, Germany), in which glucose, as a primary carbon source, is gradually provided from a soluble polysaccharide by biocatalytic degradation [85], has been successfully used for high-yield cytoplasmic and periplasmic expression of several proteins including antibody fragments [86,87,88,89]. Several studies have demonstrated that the yield of recombinant antibody fragments in *E. coli* significantly improves at growth temperature below 30 °C, likely due to the reduction of translation rate that favors proper Ig-like folding and reduces aggregation [90,91,92].

To overcome the limitations of operating parameters, a slower protein synthesis approach, named “stress minimization” has been developed with positive effects on the yield and solubility of correctly folded recombinant proteins [93]. From an experimental point of view, this approach consists of the careful management of variables such as growth temperature, inducer concentration and time point of induction, whereby growth and RPP proceed concurrently at slower rates. Stress minimization results in the increased viability of cells and process robustness [70,94]. In the so-called Design of Experiments (DoE) setting [95] the optimization of the expression of recombinant Fab and scFv fragments in stress minimization conditions has been successfully achieved through the selection of media [70,88,96,97], screening of signal peptide sequences [67,68,75,98] and optimization of co-expressing chaperone proteins [56,88,99,100,101,102]. At the transcriptional level, the concept of “codon harmonization”, a sophisticated version of the codon usage optimization, has largely improved the expression of antibodies fragments [60,103].

To overcome the variations deriving from different plasmid vectors a very recent benchmark study has introduced the use of the gene integration (GI) approach to improve the production of Fabs expressed in the *E. coli* periplasmic space in fed-batch fermentations [104]. Recently, Hausjell and coworkers [74] have reported the use of a plasmid-free expression technique for Fab antibody fragments using the BL21 (DE3) expression system. According to the “Recombineering approach”, the genes codifying for the heavy and light chains of a Fab fragment were encoded under the control of the T7 promoter and integrated into the genome at the attTN7 site [105]. They have demonstrated that in genome-integrated T7 expression systems, IPTG results in a better inductor compared to lactose in terms of cell fitness and Fab fragment productivity, as opposed to the known toxic effects of IPTG in plasmid-based T7 expression systems [106].

### 2.4. Protein Localization in E. coli

*E. coli* cells, like other Gram-negative bacteria, possess an inner and outer membrane that separates the organism into two main subcellular compartments: the cytoplasm and the periplasm. The most common choice for the production of recombinant proteins is the cytoplasm; however, the periplasmic space or the extracellular environment are more suitable when disulfide bonds are required for correct protein folding. As the disulfide bridges cannot be efficiently formed in the reducing conditions of the cytoplasm, antibody fragments are most commonly engineered with a signal sequence that directs them to the more oxidizing bacterial periplasm for proper folding. Folded fragments may further leak from the periplasm into the culture medium (extracellular localization) from which they can be purified without cell lysis. In *E. coli*’s cytoplasm, cysteine-rich polypeptides result mostly in nonfunctional aggregates. Alternative strategies including redox mutant strains with more oxidizing cytoplasm conditions and the coexpression of molecular chaperones could be employed to facilitate the correct folding also in the cytoplasmic space.

In the next sections, the most significant progress achieved in the production of Fabs and scFvs in three different compartments are gathered, analyzed, and matched according to the influence that critical parameters have on the improvement of the quality and recovery of recombinant antibody-like fragments.

### 2.5. Cytoplasmic Expression

The bacterial cytoplasm provides ample space for protein accumulation and is generally well-suited for the expression of most soluble recombinant proteins [107]. However, the production of homogenously folded disulfide-bonded proteins like antibody fragments is hampered by the cytoplasm reducing conditions and by the lack of suitable molecular chaperons. The excessive production of recombinant proteins in the bacterial cytosol often forces partially folded proteins to interact with each other resulting in protein aggregation and IB formation. The cytoplasmic reducing environment also contributes to protein misfolding and IB formation by inhibiting the intradisulfide bond formation capability.

The cytoplasm has a negative redox potential and this reducing environment is also populated by the thioredoxin–thioredoxin reductase (trxB) and the glutaredoxin–glutaredoxin reductase (gor) systems. Several enzymes like ribonucleotide reductase (RNR), methionine sulfoxide reductase (MsrA), phosphoadenosine phosphosulfate (PAPS) reductase, arsenate reductase (ArsC) and hydrogen peroxide-inducible gene activator (OxyR) continuously regenerate the active thiol sites following a catalytic cycle that is efficiently managed by these reducing pathways [108]. The use of redox-altered mutants of strains such as SHuffle and Origami and others restores an oxidizing environment in the bacterial cytoplasm, thereby their use significantly facilitates the production of soluble cytoplasmic recombinant proteins containing disulfide bonds.

Recently, it has been reported that the expression of an scFv against HER2 derived from Trastuzumab (drug bank number DB00072) in SHuffle at 30 °C resulted in enhanced solubility and a higher expression level of the molecule as compared to its expression in BL21 (DE3) at 37 °C [109,110,111,112]. The final production yield of the anti-HER2 scFv was 147 mg/L, under optimal expression conditions (24 h after induction with 0.05 mM IPTG at 30 °C, LB medium, SHuffle strain). The coexpression of molecular chaperones has also been attempted to improve the folding and stability of scFvs and Fabs into the cytoplasm [113,114,115]. Currently, several plasmids such as pG-KJE8 (dnaK-dnaJ-grpE, groES-groEL), pGr07 (groES-groEL), pKJE7 (dnaK-dnaJ-grpE), pG-Tf2 (groES-groEL-tig) and pTf16 (tig) have become commercially available for the expression of the most widely used cytoplasmic chaperone systems [116,117] such as DnaK-DnaJ-GrpE, trigger factor (TF) and GroEL-GroES. Some comparative studies have demonstrated that the levels of functional Fabs and scFvs have been largely improved in the presence of these cytoplasmic chaperones [113,114]. Recently, Liu and coworkers [102] have reported the cytoplasmic expression of a soluble and active scFv with a yield up to 12.8 mg/L. They have strategically mimicked the oxidizing environment by a combination of the SHuffle strain and by the coexpression of the chaperone proteins GroEL, GroES, DnaK, DnaJ, GrpE and trigger factor (TF) [102]. Through a systematic screening of chaperones, they have identified the GroEL-GroES system as the best performing for the preparation of scFv fragments cloned in pET28 and bearing an N-terminus hexahistidine tag. Plasmids were transformed in two Shuffle-derived strain cells, one containing pG-KJE8 and the other containing pG-Tf2 vectors, and a large-scale preparation was efficiently achieved using the SHuffle strain containing pG-KJE8 in TB medium following induction with 1 mM IPTG at 15 °C o.n. These results suggest that, since the coexpression of chaperones is a critical variable for enhancing the expression yield of soluble scFv/Fab proteins in the *E. coli* cytoplasm, there is not a universal methodology for overcoming the folding problems. Thereby, the successful combination of molecular chaperones and target proteins is identified by a trial and error process. Recently, the same research group reported the use of cyclic scFv variants to overcome the aggregation propensity mediated by interchain VH-VL interactions [35,36]. Cyclic scFvs have been obtained by both covalently connecting the N-terminus and the C-terminus using sortase A and through the split intein-mediated in vivo protein ligation techniques. Accordingly, they optimized the production of soluble cyclic scFv (2.8 mg/L) in bacterial cytoplasm by a combination of chaperone coexpression and SHuffle strain [36].

Several reports have also suggested that the use of fusion partners such as GST [118], maltose-binding protein (MBP) [119,120], small ubiquitin-related modifier (SUMO) [87,121] and thioredoxin (Trx) [122] to the target proteins, especially to scFv fragments, results in solubility-enhancing properties and in increased yields of soluble and active products. However, the fusion partners must be cleaved as big tags usually interfere with the folding of the target protein and with its activity. The entire process of tags removal is however costly and laborious and not often utilized in production processes [123].

The SUMO fusion technology has been successfully applied to the cytosolic production of Fabs in *E. coli*. The highest yield of correctly folded and biologically active SUMO-tagged Fab, 12 mg/L, was recovered from cells harvested after a 16 h growth at 30 °C post-induction with 0.5 mM IPTG using the SHuffle strain and the EnBase medium [87].

Recently, the research group of Nakano has reported the use of a small tag, referred as the SKIK sequence (serine-lysine-isoleucine-lysine), that is able to improve the cytoplasmic expression of Fab fragments in the *E. coli* SHuffle T7 Express strain using the LB medium at 16 °C for 24 h [124,125]. They also described an *ad hoc* engineered Fab fragment bearing a leucine zipper (LZ) pair at the C-termini of both the heavy and light chains, a construct named Zipbody. These extensions would enhance the chain pairing in the active form in both the *E. coli* cytoplasm expression and in the cell-free protein synthesis systems, a technology named Ecobody [124,125,126].

To overcome the limitations of *E. coli* cytoplasm expression, Gaciarz and coworkers [54,55] have developed the CyDisCo (Cytoplasmic Disulphide bond formation in *E. coli*) system. It is based on the co-expression of a catalyst of disulfide bond formation, usually a sulfhydryl-oxidase such as Erv1p, DsbB or VKOR, plus a catalyst of disulfide bond isomerization like DsbC or PDI. The CyDisCo has been exploited as an efficient route for the production of scFv and Fab fragments derived from known antibodies of different classes and from different organisms (human, mouse and humanized) in the cytoplasm of the KEIO (collection parental K12) *E. coli* strain in shake flasks [127]. The production of an anti-HER2 scFv with CyDisCo was 251 mg/L using EnPresso B media [54] which is 50.3% higher than the yield obtained without CyDisCo using only EnPresso B (167 mg/L) and 70.5% higher than that obtained in SHuffle using the LB medium (147 mg/L) [109]. Additionally, unlike the ΔtrxB/Δgor strains (SHuffle or Origami), CyDisCo, preserving the native cytoplasm environment, results also amenable to large-scale cultivation in chemically defined minimal media as demonstrated by the high-expression yield (139 mg/L) of the scFv of an IgA1 [55]. Collectively, these results highlight the enormous potential of the SHuffle strain for the production of soluble fragments of active antibodies in the *E. coli* cytoplasm, especially scFv. Its use in combination with different chaperones or fusion tags and with the Enpresso B medium, therefore, paves the way to the large-scale production of antibody fragments through cytoplasmic expression also on a lab scale [128]. With such high-expression yields in the flasks, the production of antibody fragments would not require a scale-up to bioreactors during the drug development process [129].

### 2.6. Periplasmic Expression

The *E. coli* periplasm provides the natural oxidizing conditions for disulfide bond formation and isomerization due to the presence of enzymes and specific chaperones and foldases that facilitate the production of soluble and active proteins containing disulfide bonds [130]. All proteins in *E. coli* are initially synthesized in the cytoplasm as precursors carrying a cleavable N-terminal signal sequence that directs them to the general secretion pathways at the inner membrane. Like other Gram-negative bacteria, *E. coli* exploits three main pathways for protein translocation to the periplasm: the SecB-dependent, the SRP-mediated and the twin-arginine transport (TAT) translocation pathways [131,132,133]. The SecB and SRP pathways employ the SecYEG complex, a pore in the inner membrane that transports the unfolded polypeptide chains from the cytoplasm to the periplasm.

The SecB pathway is post-translational and the polypeptide chains are translocated after the translation is complete. The SRP pathway instead is co-translational because the translocation occurs while the polypeptide chain is still being translated by the ribosome.

The third mechanism, the twin-arginine transport (TAT) system pathway, consists of a larger pore made up of the TatABC proteins, which transport the fully folded proteins into the periplasm. Proteins with slow folding rates are generally translocated via the SecB pathway, while rapidly folding proteins favor the TAT pathway. Although the TAT system has been successfully used in many cases [134] the majority of recombinant proteins (>90%) translocating to the periplasm are directed via the SecB and SRP pathways. Targeting of the polypeptide chains to the periplasm via SecB, SRP or TAT requires an N-terminal signal peptide that specifically interacts with components of the three secretory pathways. This signal peptide is then opportunely cleaved from the polypeptide chain by proteases during the translocation in the periplasm.

Once they reach the periplasm, the newly exported mature proteins are folded and assembled. Periplasmic proteins may encounter two types of protein folding catalysts: protein disulfide isomerases (Dsb proteins), which catalyze the formation of disulfide bonds, and peptidyl-prolyl isomerases (PPIase), which catalyze the cis-trans isomerization of peptidyl bonds.

**Figure 1:**
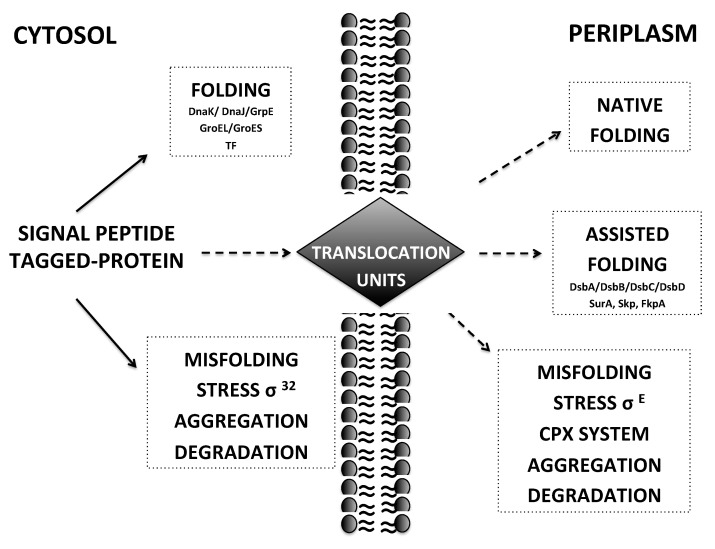
Schematic representation of optional folding and misfolding pathways for periplasmic recombinant protein.

The Dsb protein system is composed of five members: DsbA, DsbB, DsbC, DsbD, DsbG [135]. The DsbA/DsbB system assists the formation of disulfide bonds but the process may result in incorrect cysteine pairing and in the trapping of the target protein in non-native conformations. Isomerases DsbC and DsbG promote the rearrangement of the scrambled disulfide bonds assisted by the integral inner membrane enzyme DsbD, which constantly reduces these latter isomerases by transferring the electrons made available by the cytoplasmic thioredoxin. In addition, DsbA, DsbC and DsbG may also have a chaperone activity that favors the recognition and interaction with substrates necessitating disulfide isomerization [136]. The process leading to correct protein folding in the *E. coli* periplasm is further completed and checked by the activity of PPIases such as SurA, FkpA, PpiA, PpiD, Skp, and DegP (see Figure 1) [137,138,139,140]. PPI are enzymes that catalyze the cis-trans isomerization of peptidyl bonds and their activity is the rate-limiting step of the protein folding.

### 2.7. Advantages and Disadvantages of the E. coli Expression Systems

In addition to the peculiarity of providing a natural oxidizing environment that promotes the formation of disulfide bonds, the secretion of recombinant proteins in the periplasmic space offers other potential advantages. For example, the translocation reduces the exposure of the recombinant protein to host cytoplasmic proteases, reducing the degradation. Additionally, recombinant proteins are produced with a true N-terminus, thus without an N-terminal methionine. For proteins expressed in the periplasm the downstream processing is also simplified because it contains only around 4% to 8% of the natural *E. coli* cellular proteins and the outer membrane can be stripped away applying an osmotic shock or mild heat treatments. In spite of the above-mentioned benefits, the production of recombinant proteins into the *E. coli* periplasmic compartment is limited by the periplasm size and by the secretion capacity of the cell.

The periplasmic compartment accounts for less than 20% of the total cell volume. Depending on the strain, on the signal peptide used for secretion and on the protein of interest, there is a threshold of the protein amount that can be exported into the periplasmic compartment. Above a certain optimal rate of translation, secretion rates can rapidly decrease. Indeed, when the expression is too high, the translocation is slowed down by poorly exported proteins or by defective signal peptides, thereby metastable precursors may accumulate in the cytoplasm promoting inclusion bodies formation that affects protein yields and cell viability [141,142]. This excess of precursors of secretory proteins in the cytoplasm induces the up-regulation of heat shock proteins, which is mediated by the sigma factor 32 (σ32) [141], promoting inclusion bodies formation, reducing protein yields and cell viability (increased cell toxicity). Furthermore, it is not surprising that an excessive *E. coli* stress response could generate increased demand for protein folding and induce an uncharacterized metabolic burden on the cells that leads to protein misfolding and aggregation also in the periplasm. So far, the Cpx two-component system (2CST system CpxRA) and the heat shock σE pathway have been well-characterized as two regulatory transduction pathways of Envelope stress responses (ESRs) systems for preventing any perturbation in the periplasmic protein folding [143].

Another common drawback is related to the leakage of antibody fragments in the medium. The metabolic stress leads to a high accumulation of antibody fragments in the periplasm saturating the secretory machinery. This event generates a more permeable membrane structure that, after sufficient product accumulation in the periplasm, allows a higher diffusive leakage of the periplasm proteins outside the cells [86,144]. However, the optimization of extracellular secretion is becoming another valid option to produce active folded recombinant antibody fragments in *E. coli*. To overcome the issues associated with protein extracellular effusion and loss, careful optimization is required to match recombinant expression rate with the secretion capacity of the host to optimize translocation and folding efficiency. Protein secretion can be effectively modulated at the transcriptional level by modifying the promoters in the expression vector [145]. Additionally, the choice of the signal peptide sequence [146,147,148,149] and the co-expression with chaperons can affect the secretion and folding (see Figure 1).

### 2.8. Choice of Peptide Signal for Antibody Fragment Expression

Signal peptides act as zip codes marking the protein secretion pathway as well as the protein target location. The choice of the signal peptide has a strong impact on recombinant protein production rate and yield in the periplasm. Recently, Kulmala and coworkers have investigated the effect of synonymous codon pairs and mRNA secondary structures on the pelB peptide sequence for the periplasmic expression of a Fab fragment [62]. By screening a combinatorial library through ad hoc developed time-resolved fluorescence immunoassays [60], they firstly evaluated the effects of synonymous codon mutations in the n-, hydrophobic and c-region of the pelB signal sequences of the light and heavy chains cloned into a bicistronic vector under the control of a Lac promoter. Then the effects of codon usage and mRNA secondary structures were further evaluated for improving the Fab periplasmic expression. The use of an optimal nucleotide triplet coding for leucine in position 5 of the pelB sequence of the light chain resulted in a reduction of the expression level. These results confirmed that the presence of rare codons present in Sec signal peptides is not casual, but is highly important to ensure an efficient interaction of the export proteins with the components of the secretory machinery and also to prevent their degradation [150,151]. Furthermore, bioinformatic analyses related to mRNA secondary structures at the translation initiation regions of the light and heavy chains supported their role on the expression levels [152]. The reduced folding energy of the mRNA secondary structures at the translation initiation region of the light chain and the presence of rare codons in the signal peptides coincided with increased Fab expression.

Sophisticated bioinformatic tools can also be used for the *in silico* prediction of signal peptide sequences and for their cleavage positions also in bacterial amino acid sequences. These include the consolidated PrediSi platform [153] and the more recent Signal_P5 [154] and Mature P [155].

Beyond pelB, other N-terminal signal sequences derived from the outer membrane protein A (OmpA) or from alkaline phosphatase A (PhoA) have been utilized to transport antibody-like fragments to the periplasmic space of *E. coli* via the *Sec* pathway and the SRP-dependent pathway [146,147,156]. The 22 amino acids long PelB (pectate lyase B) signal sequence from *Erwinia carotovora* [157] is the most frequently used for transportation of the Fabs [88,96] and scFvs [30,101,158,159]. Recently, the expression of an scFv antibody fragment has been used as a showcase for validating the efficacy of the novel vector pSAR-2 containing the pelB leader sequence and the rhamnose-inducible expression promoter, obtaining a yield of 1.2 g/L [71]. A number of other methods and tools have been also devised for exploring the different features of signal peptides and their ability to modulate the expression of scFvs [67,68,98] and Fabs [56,60,88,90,96,99].

For instance, Kasli and coworkers [67] have evaluated the influence of the secretory pathways in scFv periplasmic recovery by comparing the use of the pelB signal peptide (directing to the periplasm via the post-translational SecB pathway) and the DsbA signal peptide (targeting to the periplasm via the cotranslational SRP route). The pelB signal sequence resulted largely superior over the DsbA signal peptide in terms of scFv solubility and cell physiology [67].

An innovative β-lactamase screening system for the optimization of signal peptides has been developed using pelB as scaffold [98]. In this work, a preliminary screening production of the scFv 13R4 in the periplasm driven by the arabinose-inducible pBAD promoter (pLBAD2 vector) using STII, DsbA and PelB signal peptides was performed. pelB was selected and used as the starting point for constructing both an epPCR library (random mutagenesis signal peptide library) and a chemically synthesized (CS) peptide library. According to the signal related to β-lactamase activities, two new peptides were selected from the libraries as improving the periplasmic production of active 13R4 by ~40% compared to the expression obtained using the wild type pelB [98].

Kumar and coworkers have obtained a yield of 25 mg/L of a periplasmic Fab fragment in shake flasks by using the rhamnose-inducible promoter (rhaBAD) and the *mal* and pelB secretion sequences for the heavy and light chains, respectively. The highest biomass and expression was obtained using BL21 (DE3) *E. coli* cells induced with 50 mM rhamnose at 30 °C for 8 h in the Champion medium [96]. A similar periplasmic recovery (30 mg/L) of functional Fab was reported using pelB and induction with 0.1 mM IPTG at 30 °C o.n. when coexpressed with the DsbA/C chaperones in shake flasks [88]. In another study, a recovery of 10 mg/mL of highly immunoreactive Fabs was obtained by using pelB, the BL21 strain and the SB medium in combination with a harmonizing DNA approach [60].

In another study, the use of the heat-stable enterotoxin II (STII) signal peptide led to an expression yield of 332 mg/L of soluble Fab. Here, a gross nitrogen flow was supplied (6.91 g/L) in a 5L scale fermentation at 25 °C using an ad hoc engineered W3110 (ilvG^+^/^+^ ΔphoA) *E. coli* strain. These results indicate that supplementing a nitrogen source at low temperature is critical for Fab productivity in *E. coli* fermentations [90]. Of note, the alkaline phosphatase (phoA) promoter and the heat-stable enterotoxin II (STII) leader sequence have been also described as a positive combination to facilitate the *E. coli* extracellular production of Fab fragments [160].

Karyolaimos and coworkers [68] have successfully demonstrated that a combinatorial screening of different signal peptides in a titratable system that tunes protein production rates was a valuable and effective approach to enhance scFv recombinant production yields in the periplasm of *E. coli*. The gene encoding for an scFv bearing at the C-terminus a His6-tag was fused to DsbAsp, Hbpsp, OmpAsp, and PhoAsp signal peptides and, in turn, inserted into the rhamnose promoter-based expression vector pRha. This enabled tuning of the protein production rates by varying the rhamnose concentration and avoiding the saturation of the Sec-translocon capacity. For the expression, plasmids were transformed into the *E. coli* strain W3110Δ rha Δlac [161]. The highest periplasmic production yield of an scFv was 0.2 g/L of culture and was achieved using the OmpA signal peptide, LB medium and induction with 100 μM rhamnose at 30 °C in a shake flask [68]. Additionally, Fab fragments bearing the OmpA signal peptide were efficiently produced as soluble periplasmic products by coexpression with DnaK/DnaJ/GrpE in shake flasks using the LB medium and inducing with 0.1 mM IPTG at 25 °C for 8 h [99]. When the same Fab was coexpressed with DsbC in fermentation scale using the ad hoc genetically modified *E. coli* strain deficient in the Tail specific protease (Tsp) and SRP, an optimal recovery of 2.4 g/L was reached [56]. Recently, the OmpA-leader sequence (MKKTAIAIAVALAGFATVAQA) was also selected as the best in plasmid-free expression systems (GI) for the production of a functional Fab fragment [74,104].

### 2.9. Enhancement of Fab/scFv Secretion into the E. coli Periplasm by Coexpression with Chaperones

The periplasmic localization of several proteins’ folding factors and chaperones able to catalyze the proper assembly and folding of functional Fab and scFv antibody fragments has been largely studied. In particular, the correct folding of scFv and Fab fragments has been found to be highly dependent on the activity of PPIases [162]. Following the formation of the intrachain disulfide bonds of variable and constant domains, peptidyl-prolyl cis-trans isomerization reactions drive the folding of Fabs into native conformations. The PPIase activity favors the adoption of correct Ig-like folds playing a crucial role in the prevention of misfolding/aggregation events of antibody fragments. Notably, the kappa light chain variable domains (Vκ) contain two conserved prolines in the cis conformation at positions L8 and L95 (Kabat numbering), unlike the heavy-chain variable (VH) and lambda light chain variable (Vλ) antibody domains [163]. Pioneering studies reported by Plückthun and coworkers [164,165] on the aggregation properties of scFv fragments, demonstrated that the slow isomerization of the peptide bond preceding Pro-L95 is important because it must be in the cis conformation for the formation of the native VH/VL interface. A cis-trans isomerization at Pro-L95 is a rate-limiting step in the folding of the Vκ domains and is essential for the VL/VH docking and, therefore, for the adoption of native protein conformations. The lack of proper peptidyl-prolyl isomerization activity can drive the formation of off-pathway folding intermediates that promote aggregation. Later, the same group also reported that the coexpression of FkpA (a periplasmic PPIase of *Escherichia Coli*) resulted in a significant improvement of secretion into the bacterial periplasm of functional scFv fragments containing either Vκ chains, which contain cis-prolines, or Vλ chains which do not contain cis-prolines, suggesting that it has both molecular chaperone and PPIase enzymatic activities [166]. Several groups have attempted with varying degrees of success to improve the bacterial production of antibody fragments also by coexpressing them with molecular chaperones or folding catalysts [122,167,168].

Currently, among the different periplasmic chaperones and/or folding catalysts, the DsbA and DsbC thiol-disulfide oxidoreductases, and two PPIases with chaperone activity, FkpA and Skp, result the most used in coexpression settings. Beyond the basic concept of filling up with chaperones their natural cellular compartments, several groups have also adopted an “interchangeable approach” where the effect of chaperones, both cytoplasmic and periplasmic, has been evaluated in an interchangeable manner both in terms of site of action (making the periplasmic ones devoid of the signal sequence) and in terms of localization of the production of the recombinant antibody fragments.

For instance, Dariushnejad and coworkers have reported that the coexpression of DnaK/DnaJ/GrpE (DnaKJE) results in a 2.5-fold increase in the periplasmic expression level of an anti-TNF-α Fab in shake flasks, using LB medium and BL21(DE3) strains [99,100]. No relevant improvements were detected using the Shuffle strain. Following comparative studies, the authors found that also other chaperones have the ability to increase the solubility of Fab fragments but the DnaKJE chaperone system resulted in the best in terms of activity. This evidence sheds light on the importance of evaluating the activity of soluble antibody fragments to confirm the correct Ig-like folding. As already mentioned above in the cytoplasmic expression paragraph, it has been reported that the coexpression of the periplasmic chaperone DsbC significantly increased the Fab antibody fragment expression in the bacterial cytoplasm using the Origami (DE3) strain [113]. Taken together these data suggest that DsbC overexpression in the cytoplasm exerts a positive effect on the solubility of cytoplasmic proteins [113] not on the recombinant proteins that are targeted to the periplasmic space [99].

The effect of coexpression of cytoplasmic chaperones such as GroEL, DnaJ, Tig, GroES, DnaK and GrpE (see Figure 1) has been evaluated also on the expression of an anti-CD20 human scFv [27]. This scFv fragment was cloned into the pET22b vector containing the pelB sequence and was systematically cotrasformed with five commercial plasmids containing different chaperone combinations in BL21 (DE3). Cells were cultured in LB broth and the enhancement of expression was evaluated after induction with 1 mM IPTG for 4 h at 25 °C. Importantly, the coexpression of the pKJE7 plasmid containing GrpE/DnaK/DnaJ had the highest outcome (up to 50%) on solubility compared to other chaperone combinations. Similarly, Sonoda and coworkers [122,167] have reported that the coexpression of DnaKJE with GroELS had negative effects on recombinant protein production in the cytoplasm thus suggesting that there is no cooperativity between GroELS and DnaKJE chaperone systems.

In another report, the cytoplasmic variant of chaperone FkpA (cyt-FkpA) was sucessfully used for improving the periplasmic expression of a Fab fragment [169]. The expression was optimized using commercial TG1 cells harboring the Fab and chaperone plasmid constructs in 2xYT, growth media containing 0.2% arabinose (w/v), and inducing with 1mM IPTG overnight at 30 °C. According to the experimental evidence, authors speculated that the cyt-FkpA has an instrumental cytoplasmic role that improves folding and Fab assembly. It indeed isomerizes key prolines of the kappa light chains prior to the periplasmic export thus preventing a folding bottleneck and favoring the translocation of the heterologously expressed polypeptide chains into the oxidizing periplasmic environment. The strategy improved the levels of soluble periplasmic Fab from 0.4–2.5 mg/L to 3.5–14.2 mg/L.

The synergistic effect of DsbA/DsbC has been next successfully assessed as an effective way to improve the soluble expression of Fabs [56,88] and scFvs [170] in the *E. coli* periplasmic space. In this regard, Rodriguez and coworkers have reported a significant improvement in functional Fab expression into the *E. coli* periplasm (30 mg/L) as a result of its coexpression with the wild type periplasmic DsbA/C [88]. In this case, the BL21 strain harboring the pLac-Fab3F3 and the pBAD-DsbA plasmids was cultivated in EnBase medium [85]. Similarly, Ellis and coworkers developed a novel approach based on the coexpression of Fab fragments with DsbC in ad hoc engineered *E. coli* strain (Tsp spr strains) achieving a very high yield of periplasmic Fab approaching over 2.4 g/L in fermentation scale after 40 h post-induction [56]. The Fab expression was achieved using the pTTO plasmid [171] containing a strong IPTG-inducible tac promoter and the OmpA signal peptide. The lack of the Tsp protease and its extragenic suppressor *spr* in *E. coli* host cells resulted in a recovery of the “wild type” cell viability thus favoring the expression of the Fab and its correct folding in the presence of the Dsb chaperone.

Sun and coworkers reported a yield of 33 mg/L of a soluble and active scFv in shaking-flask cultures by using BL21 (DE3) cells, 2xYT media and following induction with 0.2 mM IPTG for 4 h at 30 °C [170]. For the periplasmic expression, the scFv was cloned into a commercial pET-26b(+) vector whereas Dsb proteins were cloned into the pACYC-Duet-Ara plasmid, a modified version of the pACYC-Duet-1 plasmid (Novagen), where two T7 promoters are replaced by an arabinose-inducible araBAD promoter. These results suggested that the pACYC-Duet-Dsb coexpression vector might be a useful tool for the production of soluble and functional scFv antibody fragments.

In another study, the coexpression of an scFv with the periplasmic chaperones FkpA and Skp significantly improved the cytoplasmic solubility of the scFv and cell viability [168,172].

### 2.10. Extracellular Secretion

A third option and collateral way to produce recombinant antibody fragments is their recovery directly from the surrounding *E. coli* culture medium. In general, a target protein fused to an N-terminal secretion tag can be recognized and translocated by the Sec machinery to the periplasm but it could further cross the outer membrane reaching the extracellular medium giving rise to a secretory protein. Extracellular protein expression holds several advantages over intracellular production such as the more oxidative and ample environment for effective protein folding, a higher titer of recombinant protein expression, and straightforward downstream purification process. The extracellular expression also enables the direct harvesting of proteins from the culture supernatant, sparing the procedures of cell lysis and reducing the process-related impurities, such as host cell DNA and endotoxins. Furthermore, extracellular secretion can prevent the accumulation of insoluble inclusion bodies in the cytosol or periplasm as well as the toxic effects exerted by some target proteins on the host upon their intracellular expression. The extracellular secretion can be successfully achieved by optimizing the induction starting point and by adding chemical agents that promote outer cell membrane permeability [131,148,173]. To date, the supplementation of Triton X-100 or glycine to the culture medium facilitates the extracellular secretion of target proteins from *E. coli* cells in periplasmic expression settings. Indeed, while glycine induces the swelling of *E. coli* cells and enlargement of the periplasmic space by interfering with the synthesis of peptidoglycans, Triton X-100 disrupts the integrity of the outer membrane [174]. In this way, a functional recombinant scFv has been efficiently recovered (2.86 mg/L) from the extracellular medium by adding 0.25% Triton X-100 [159]. An experimental setting for the efficient secretion of Fab fragments in *E. coli* in shake flasks has been recently described by Luo and coworkers [160]. They have demonstrated that the use of the alkaline phosphatase promoter (phoA) in combination with the heat-stable enterotoxin II (STII) signal peptide (phoA-STII system) is a promising strategy for the extracellular production of Fab fragments, reaching up to 10 mg/L [160]. The authors carefully assessed and compared the effects of promoters, *E. coli* strains and signal peptides on the extracellular expression of a panel of five recombinant Fab fragments. Of interest, they found that the secretion efficiency of the STII signal peptide could be further improved by the coexpression of TolC, the major efflux pump in gram-negative bacteria. The ST signal peptide is translocated across the outer membrane via the TolC/MacAB system [175,176]. Therefore, the overexpression of TolC dramatically enhanced the Fab recovery from the extracellular medium.

**Figure 2:**
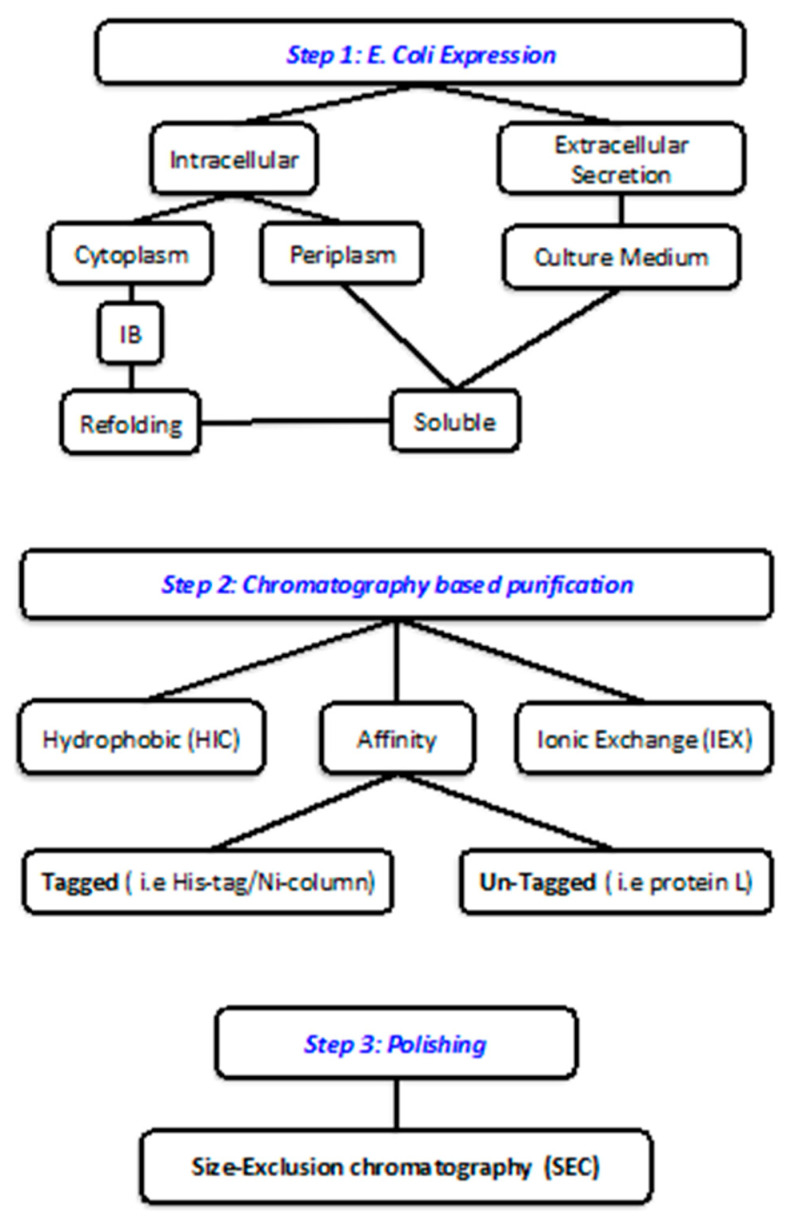
Three step procedure for obtaining purified recombinant antibody fragments following the expression in *E. coli*.

Given the undeniable advantages of the production of recombinant protein directly in the extracellular space, several technology platforms have been developed for the large-scale production of these biotherapeutics in *E. coli* supernatants. A graphical overview of the general procedures adopted for obtaining antibody fragments following expression in *E. coli* is reported in Figure 2.

### 2.11. New Platforms and E. coli-based Cell-Free Expression System

Currently, the *E. coli*-based ESETEC Wacker’s secretion technology is one of the most efficient and cost-effective platforms. It relies on the use of the K12 modified strain that enables yields up to 4.0 g/L for Fab fragments and 3.5 g/L for scFvs into the fermentation broth (https://www.wacker.com/cms/en-us/products/brands/esetec/esetec.html).

The RiboTite gene expression control is another innovative technology for protein expression in the cell supernatants (https://gtr.ukri.org/resources/contact.html). It is based on the dual transcriptional–translational gene expression control where a dual Lac-operator/repressor promoter and an orthogonal riboswitch modulate both T7 RNAP and the GOI. In general, a riboswitch is a segment in a messenger RNA that folds into intricate structures that prevent the expression of target genes by interfering with the translation. The binding of an effector molecule induces a conformational change that post-transcriptionally regulates the protein expression. The Dixon lab has developed this system by combining the pETORS expression vector (pET vector engineered with orthogonal riboswitch sequence (ORS) sequence) and ad hoc engineered strains named BL21(IL3) and BL21(LV2) [75,177]. In addition to the IPTG-induced translational control, an orthogonal riboswitch sequence (ORS) controls at the transcriptional level the expression of both chromosomal copies of T7 RNAP and the episomal copies of the recombinant gene of interest. The riboswitch sequence, a modified version of the adenine-sensing A-riboswitch from *Vibrio vulnificus*, is controlled by pyrimido-pyrimidine-2,4-diamine (PPDA) [178], thereby the expression of the target gene occurs only in the presence of both IPTG and PPDA, which effectively reduces the leaky expression to almost undetectable levels.

Batavia Biosciences has developed its own platform technology for cost-effective protein production in *E. coli*, called SCOPE^®^ technology (https://www.bataviabiosciences.com/scope-technology). SCOPE enables the generation of proteins expressed in *E. coli* strains with high yields and tight control of protein expression. In particular, by using the pSAR2 plasmid with a rhamnose promoter, the scFv PGT135 antibody fragment was successfully produced in the periplasmic space. Amounts up to about 1.2 g/L of the biologically active scFv PGT135 were recovered [71].

In recent years, the cell-free protein synthesis systems (CFPS) have been used as an alternative approach to overcome the limitations associated with cell-based expression methods. The well-known and innovative Ecobody technology [179] enables an efficient and cost-effective production of functional proteins including monoclonal antibodies and related fragments [76,180,181,182]. Taking advantage of the CFPS systems, *E. coli* extracts are used to produce Fabs derived from single B cells. The Zipbody and the SKIK peptide tag technologies have been developed to improve the *de novo* synthesis of soluble and functional Fab fragments [124,126]. Recently, the Ecobody technology has been successfully employed to produce two Fab fragments needed to set up a rapid ELISA assay for the detection of swine influenza virus [183].

Furthermore, Sutro Biopharma has recently developed a novel and flexible Xpress CF platform (Cell-free platform) (https://www.sutrobio.com/technology/xpress-cf/) for the expression of multispecific antibody and antibody–drug conjugates (ADC) in the CFPS modality. The process produces single proteins at g/L yields in 8–10 h at any scale. In particular, the XpressCF+™ technology, through the insertion of non-natural amino acids, provides therapeutic proteins with site-specific conjugation groups. For example, the technology has been used to produce the Sutro’s clinical ADC products STRO-001 and STRO-002, in which a cytotoxin is conjugated to an antibody containing non-natural amino acids (STR001—Clinical Trial: NCT03424603. Available online: https://clinicaltrials.gov/ct2/show/NCT03424603).

## 3. Antibody Fragments as Biotherapeutics and Theranostic Agents

Thanks to their improved pharmacokinetics and their structural and functional flexibility, the three main antibody surrogates, single-domain antibodies (dAbs), scFv and Fabs, are continuously developed and reformatted into bispecific/multi-specific molecules or cytotoxic/radioactive drug carriers to achieve a desired biological efficacy and for multiple clinical applications [1]. In Table 1, most of the classic and ad hoc engineered antibody fragments currently under clinical development or FDA-approved are summarized. We briefly review here five of these that are recombinantly produced in *E. coli* and used for therapeutic intervention.

Certolizumab pegol (CIMZIA^®^) is a PEGylated Fab’ fragment of a humanized anti-TNFα monoclonal antibody. It was developed and manufactured by UCB Pharma and first approved by the FDA in 2008 for treating rheumatoid arthritis. The drug received new therapeutic indications on 28 March 2019 (https://www.drugbank.ca/drugs/DB08904#reference-A176606).

Ranibizumab is a recombinant humanized Fab fragment derived from the parent full-length monoclonal antibody Bevacizumab (Avastin^®^). It reduces angiogenesis by blocking the activity of the vascular endothelial growth factor A (VEGF-A). Ranibizumab is marketed under the trade name Lucentis® and is indicated for the treatment of macular edema after retinal vein occlusion, age-related macular degeneration (AMD wet), and diabetic macular edema (https://www.drugbank.ca/drugs/DB01270).

Brolucizumab, whose trade name is Beovu^®^, is a humanized scFv fragment that acts as vascular endothelial growth factor (VEGF) inhibitor, reducing the proliferation of endothelial cells, vascularization of the tissue, and permeability of the vasculature. It is approved for the treatment of neovascular age-related macular degeneration (wet AMD). Brolucizumab was granted FDA approval in October 2019 (https://www.drugbank.ca/drugs/DB14864).

Caplacizumab (trade name Cablivi^®^) is a humanized sdAb immunoglobulin anti-von Willebrand factor consisting of two identical humanized variable domains genetically linked by a three-alanine linker. Capacizumab is approved for the treatment of adults experiencing episodes of acquired thrombotic thrombocytopenic purpura (aTTP) in conjunction with plasma exchange and immunosuppression in patients 18 years or older. Caplacizumab was developed by Ablynx (a Sanofi company) and FDA-approved on 6 February 2019. The drug was previously approved in the EU in October 2018 as a combination therapy with plasma exchange and immunosuppression. (https://www.drugbank.ca/drugs/DB06081).

Moxetumab pasudotox (MxP), also named BL22, was initially referred to as an scFv fragment derived from the monoclonal antibody RFB4 which specifically binds to CD22 (a lineage-restricted B cell antigen). Then, it has been used to generate a recombinant immunotoxin in which an affinity optimized and stabilized Fv segment has been fused by a disulfide bond to the Pseudomonas exotoxin A (PE38) which has no cell-binding portion. The related drug LUMOXITI™ was developed by Astra Zeneca and FDA-approved on September 13, 2018 (https://www.drugbank.ca/drugs/DB12688).

In recent years, antibody fragments have also achieved encouraging successes as theranostic agents contributing to the development of new personalized and more effective medicine. Their tunable pharmacokinetic properties together with the unique ability to detect with high-affinity and specificity biomarkers in vitro and in vivo, making them excellent agents for tumor imaging [184,185].

An ever-growing number of arrays of antibody fragments serve also as vectors and targeting moieties in “active targeted drug delivery systems” for tumor homing through Antibody-Drug Conjugates (ADC) [186], for reducing the radiation-related toxicity of radioimmunoconjugates used in radioimmunotherapy (RIT)) [187], for nanomedicines applications [188] and also more recently for addressing CAR T cells [189,190].

## 4. Conclusions

The ever-increasing applications of antibody-based molecules as both therapeutic agents and key reagents for advanced diagnostic investigations have greatly expanded the demand of these crucial classes of molecules and the need for their production in high yield and purity. While the preparation of whole antibody molecules requires eukaryotic expression systems, antibody fragments like Fabs, scFvs and other similar surrogates that lack the glycosylation, can be conveniently prepared in *E. coli* backgrounds. The recent evolution of *E. coli* expression systems reinforces the use of this easy and cheap host-microorganism for an advisable production of antibody fragments in recombinant form. In Table 2, we report an updated list of antibody fragments described so far and the expression conditions utilized for their production, including expression localization (periplasmic, cytoplasmic), the type and format of antibody fragment, the vectors used, the inductor, the temperature and time of expression, the strain, the medium, the use of chaperones and the overall recovery. As explained in this review and reported in Table 2, during the last years, huge efforts have been done to adapt at best the *E. coli* machinery to the production of these “magic bullets” molecules, and a contribution has been also provided by structural biology and bioinformatics in addition to advanced molecular genetics, basic biology and chemical biology. Notable progress gas also emerged from the ever-increasing understanding of the unique structural features of the Ig-like domains as obtained by X-ray crystallography and accurate homology modeling studies. The structural knowledge of such basic units indeed provides a relevant contribution in the construct design for tuning the physicochemical and affinity properties and to improve the stability, the efficacy and the clinical potential of antibody-like molecules. Despite the high degree of similarity between the different components of this class of proteins and the growing availability of innovative strategies and robust tools, such as oxidizing mutant strains or plasmids for the overexpression of chaperones and foldases, and new growth media, it is evident that there is not a universal *E. coli*-based methodology for their efficient production, therefore, a trial and error optimization process is necessary for the determination of the successful experimental settings and to achieve scalability of antibody fragment expression processes at an industrial level.

## Figures and Tables

**Table 1:** Antibody fragments and formats under clinical development and FDA-approved. Sources: European Medicines Agency public assessment reports, United States Food and Drug Administration (drugs@fda), The international ImMunoGeneTics Information System® (www.imgt.org/mAb-DB/index), Animal Cell Technology Industrial Platform (www.actip.org).

Application [Radiolabeled/Conjugated/Fused]	International Nonproprietary Name	Common Name	Receptor Identification (Species)	Clinical Indication	Development Status (NCT Number)
Therapeutic	Abciximab	c7E3	Fab-G1-kappa [Chimeric]	Acute myocardial infarction [191]	Phase III [NCT00299377]
				Antiplatelet prevention of blood clots in the setting of high risk percutaneous transluminal coronary angioplasty [192]	Phase M FDA Approval, 1994
				Refractory unstable angina when percutaneous coronary intervention is planned [193]	Phase M FDA Approval, 1997
				Acute coronary syndrome (ACS) [194]	Phase IV [NCT00133003]
Therapeutic	Abrezekimab	CDP7766, UCB-4144, UCB4144, VR-942	Fab-G1-kappa [Humanized]	Asthma [195]	Phase I [NCT02473939]
Therapeutic [conjugated with pegol]	Alacizumab pegol	CDP791, g165 DFM-PEG	di-Fab’ [Humanized]	Cancers [196]	Phase II [NCT00152477]
Therapeutic [fused with CD28 (COTM-CY1) - CD247 (CY2) (1:1)]	Axicabtagene Ciloleucel	Autologous T cells transduced with FMC63 scFv-28Z CAR (FMC63 scFv-CD28-CD247 (CD3Z), KTE-C19, PG13-CD19-H3, FMC63 CD28z, Axi-cel)	scFv-kappa-heavy [Chimeric]	Diffuse large B cell Lymphoma [197]	Phase M FDA Approval, 2017
				Acute lymphocytic leukemia [198]	Phase I/II
				Follicular lymphoma [199]	Phase II
Therapeutic [fused with Homo sapiens IL2 (interleukin 2, IL-2) Pr21-153 (100%) (1:1), noncovalent dimer]	Bifikafusp alfa	L19-IL-2, L19-IL2, L19IL2	scFv-heavy-kappa [Homo sapiens]	Solid Tumor [200]	Phase I/II [NCT02086721]
				Metastatic Melanoma [201]	Phase III [NCT02076633]
Therapeutic	Blinatumomab	AMG103, BITE MT-103, MEDI-538, MT103, bscCD19xCD3	(scFv-kappa-heavy)-(scFv-heavy-kappa) [Mus musculus]	B cell Non-Hodgkin Lymphoma [202]	Phase II [NCT02910063]
				Diffuse large B cell Lymphoma [203]	Phase II [NCT01741792]
				B cell acute lymphoblastic leukemia [204]	Phase M FDA approval, 2014
Therapeutic	Brolucizumab	ESBA-1008, ESBA1008, RTH258	scFv-kappa-heavy [Humanized]	Neovascular Age-related macular degeneration [205]	Phase III [NCT03930641]
Therapeutic	Caplacizumab	ALX-0081, PMP12A2h1-linker AAA-PMP12A2h, caplacizumab-yhdp	VH–VH [Humanized]	Acquired Thrombotic thrombocytopenia purpura [206]	Phase M FDA approval, 2019
Therapeutic	Cibisatamab	CEA TCB, CEA-TCB, RG-7802, RG7802, RO-6958688, RO6958688	IgG1 - kappa/lambda with domain crossover, trivalent [Humanized]	Colorectal cancer [207]	Phase I [NCT03866239]
Therapeutic [conjugated with pegol]	Certolizumab pegol	CDP870, PHA-738144	Fab’-G1-kappa [Humanized]	Crohn’s disease [208]	Phase M FDA approval, 2008
				Psoriasis [209]	Phase III [NCT02326298]
				Rheumatoid arthritis [210,211]	Phase M
				Ankylosing spondylitis	Phase M
				Psoriatic arthritis	Phase M
				Juvenile Idiopathic Arthritis	Phase III [NCT01550003]
				Interstitial cystitis [211]	Phase III [NCT02497976]
Therapeutic [fused with GAA (glucosidase alpha, acid, lysosomal alpha-glucosidase) (Pr67-952) (1:2) Enzyme substitute]	Clervonafusp alfa	VAL-1221	F(ab’)2-G1-kappa [Humanized]	Glycogen storage disease type II (GSD-II, Pompe disease [212]	Phase I/II [NCT02898753]
Therapeutic [conjugated with pegol]	Dapirolizumab pegol	CDP7657	Fab’-G1-kappa [Humanized]	Systemic lupus erythematosus [213]	Phase II [NCT02804763]
Therapeutic [fused with Ricinus communis ricin A]	Dorlimomab aritox	4197X-RA, MDX-RA (ricin A chain) immunotoxin	F(ab’)2-nd-nd [Mus musculus]	Secondary cataract [214]	Phase III
Therapeutic	Efgartigimod alfa	ARGX-113, ARGX113	Fc-gamma1 [Homo sapiens]	Myasthenia Gravis [215]	Phase II [NCT02965573]
Therapeutic	Faricimab	RG7716, RO6867461	IgG1-kappa -lambda with half-IG VL-CH1/VH-CK crossover [Homo sapiens Humanized]	Neovascular Age-related macular degeneration	Phase II [NCT02484690]
				Diabetic macular edema [216]	Phase II [NCT02699450]
Therapeutic	Flotetuzumab	MGD-006, MGD006, RES234, S80880	V-Lambda-VH _ V-Kappa-VH’ [Mus musculus Humanized]	Acute myeloid leukemia	Phase I [NCT04158739]
				Myelodysplastic syndromes	Phase I [NCT02152956]
Therapeutic	Gancotamab	MM-302	scFv-heavy-lambda [Homo sapiens]	Breast Cancers [217]	Phase I [NCT01304797]
Therapeutic	Glenzocimab	ACT-017, ACT017	Fab-G1-kappa [Humanized]	Ischemic stroke [218]	Phase I/II [NCT03803007]
Therapeutic	Gontivimab	ALX-0171, VR-465	VH-VH-VH [Lama glama]	Respiratory Syncytial Virus Lower Respiratory Tract Infection [219]	Phase II [NCT02979431]
Therapeutic	Gremubamab	MEDI3902	[VH-CH1-scFv-VH-VK-h-CH2-CH3_L-kappa]2 [Homo sapiens Humanized]	P. Aeruginosa nosocomial pneumonia [220]	Phase I [NCT02255760]
Therapeutic [conjugated with pegol]	Lulizumab pegol	BMS-931699	V-kappa [Humanized]	Lupus [221]	Phase II [NCT02265744]
Therapeutic	Lutikizumab	ABT-981	[VH-VH’-H-Gamma1-VL-VL’-C-kappa] – dimer [Humanized]	Osteoarthritis [222]	Phase II [NCT02087904]
Therapeutic [fused with Pseudomonas aeruginosa exotoxin A]	Moxetumomab pasudotox	CAT-8015, GCR-8015, HA22, moxetumomab pasudotox -tdfk	Fv-disulfide stabilized [Mus musculus]	Chronic lymphocytic leukemia [223]	Phase I [NCT01030536]
				Hairy cell leukemia [224]	Phase M FDA approval 2018
				Acute lymphoblastic leukemia [225]	Phase I [NCT00659425]
Therapeutic [fused with Staphylococcus aureus enterotoxin E SEA/E120 superantigen (synthetic)]	Naptumomab estafenatox	ABR-217620, ANYARA, TTS CD3	Fab-G1-kappa [Mus musculus]	Renal cell carcinoma [226]	Phase III [NCT00420888]
				Nonsmall lung carcinoma [227]	Phase I [NCT00056537]
Diagnostic	Nofetumomab Merpentan	Carcinoma- Associated antigen	Fab fragment [Mus musculus]	Diagnostic imaging of small- cell lung cancer [228]	Phase M FDA approval 1996
Therapeutic	Onartuzumab	MetMAb, OA-5D5, OA5D5, PRO 143966	Fab-G1-kappa-[Fc-G1] [Humanized]	Metastatic Colorectal Cancers [229]	Phase II [NCT01418222]
				Solid tumors [230]	Phase III [NCT02488330]
Therapeutic [fused with TNF (tumor necrosis factor (TNF) superfamily member 2, TNFSF2, TNF-alpha, TNFA]	Onfekafusp alfa	L19TNF, L19TNF-alpha	[scFv-heavy-kappa - TNF (tumor necrosis factor (TNF) superfamily member 2, TNFSF2, TNF-alpha, TNFA)] –trimer [Homo sapiens]	Melanoma [231]	Phase II [NCT02076633]
				Solid tumors [232]	Phase I/II [NCT01253837]
Therapeutic [fused with Pseudomonas aeruginosa exotoxin A]	Oportuzumab monatox	VB4-845	scFv-kappa-heavy [Humanized]	Bladder Cancer [233]	Phase II [NCT00462488]
Therapeutic	Otlertuzumab	TRU-016	VH-V-kappa-CH2 -CH3 [Humanized]	Chronic lymphocytic leukemia [234]	Phase I/II [NCT01188681]
				Non-Hodgkin’s lymphoma [235]	Phase I [NCT00614042]
Therapeutic	Romilkimab	SAR156597, huTBTI3_2_1	[VH-H-Gamma4_VL-L-kappa]–dimer [Chimeric Humanized]	Idiopathic pulmonary fibrosis [236]	Phase II [NCT01529853]
Therapeutic	Solitomab	AMG 110, AMG-110, MT110	(scFv-kappa-heavy)-(scFv-heavy-kappa) [Mus musculus]	Systemic sclerosis [237]	Phase II [NCT02921971]
Therapeutic	Sonelokimab	M-1095, MSB-0010841	VH-VH’-VH, trivalent [Humanized Vicugna pacos (alpaca)]	Psoriasis [238]	Phase I [NCT02156466]
Therapeutic [fused with CD8A (COTM) - TNFRSF9 (CY1) - CD247 (CY2) (1:1)]	Tisagenlecleucel	Autologous T cells transduced with FMC63 scFv-8A-F9Z CAR (FMC63 scFv-CD8A-TNFRSF9-CD247 (CD3Z), CART19, CTL019, tisagenlecleucel-T)	scFv-kappa-heavy [Chimeric]	Diffuse large B cell Lymphoma [239]	Phase I [NCT03630159]
				Acute lymphocytic leukemia [240]	Phase M FDA approval, 2018
Therapeutic	Vanucizumab	RG-7221, RG7221, RO5520985	IgG1-kappa-lambda with half-IG VL-CH1/VH-CK crossover [Humanized]	Solid tumors [241]	Phase I [NCT01688206]
Therapeutic	Vibecotamab	XmAb14045	half-IG G1-kappa/scFv-h-CH2-CH3 [Chimeric]	Acute Myelogenous Leukemia, B cell Acute Lymphoblastic Leukemia, Blastic Plasmacytoid Dendritic Cell Neoplasm, Chronic Myeloid Leukemia, Blast Crisis [242]	Phase I [NCT02730312]
Therapeutic	Vobarilizumab	(20A11-9mer-ALB11), ALX-0061	VH-VH’ [Humanized]	Rheumatoid arthritis [243]	Phase II [NCT02518620]
				Systemic lupus erythematosus [244]	Phase II [NCT02437890]
Therapeutic	Zanidatamab	ZW-25, ZW25	[H-Gamma1_L-kappa]_scFv-VK-VH-h-CH2-CH3 [Humanized]	HER2+/HR+ Breast Cancer [245]	Phase II [NCT0422427]
				HER2-expressing Gastroesophageal Adenocarcinoma [246,247]	Phase II [NCT03929666]

**Table 2:** Summary of most relevant reported experimental settings for the production of Fab and scFv recombinant fragments in different *E. coli* compartments.

EXPRESSION	Ab-FRAGMENT	VECTOR	INDUCTOR	TEMP.	TIME	*E. coli* Strain	Medium	Chaperone	Recovery	Reference.
**CYTOPLASMIC**	Fab	pET23 modified	0.5 mM IPTG	30 °C	16 h	KEIO collection parental K12 *E. coli* strain [127]	Enpresso B	CyDisCo	3–50 mg/L	[54]
	scFv	pET23 modified	0.5 mM IPTG	30 °C	16 h	KEIO collection parental K12 *E. coli* strain [127]	Enpresso B	CyDisCo	4–271 mg/L	[54]
	scFv	pET22b	0.05 mM IPTG	30 °C	24 h	Shuffle	LB		147 mg/mL	[109]
	scFv	pET28b	1 mM IPTG	15 °C	48 h	Shuffle	TB	pG-KJE8 pG-Tf2	1–12.8 mg/ml	[102]
	Cyclic scFv	pET28b	1 mM IPTG	15 °C	48 h	Shuffle	TB	pET21-FKPB12 pG-KJE8	2.8 mg/mL	[36]
	Fab	pET28 modified	0.5 mM IPTG	30 °C	16 h	Shuffle	EnBase	SUMO fusion protein	12 mg/L	[87]
**PERIPLASMIC**	Fab	pelB/pLAC	0.1 mM IPTG	30 °C	on	BL21	EnBase	pBAD/ DsbA0.2% arabinose	30 mg/L	[88]
	Fab	pelB/pLKO4 β-lattamase	1 mM IPTG	26 °C	3 h	BL21	SB		10 mg/L	[60,62]
	Fab	Omp/pET22b	0.1 mM IPTG	25 °C	8 h	BL21(DE3)	LB	pKJE7 DnaK-dnaJ-grpE		[99]
	Fab’	Omp/pK03 tac promotor pTTO vector	0.2 mM IPTG	30 °C	40 h	W3110 (ΔTsp, spr)	SM6G medium	DsbC	2.4 g/L Fermentation	[56]
	Fab	mal and pelB/pD881	50 mM l-rhamnose	30 °C	8 h	BL21(DE3)	Champion medium		25 mg/L	[96]
	Fab	phoA/STII	FRT-method	30 °C	12 h	W3110 (ilvG+/+ ΔphoA)	YS medium		332 mg/L Fermentation	[90]
	scFv	pRha67K/Omp	0.1 mM l-rhamnose	30 °C	16 h	W3110 (Δ rha Δlac)	LB		0.2 mg/mL	[68]
**PERIPLASMIC?**	scFv	pelB/pSAR2	15 mM l-rhamnose	25 °C	48 h	BL21(DE3)	TB		1.2 g/L	[71]
	scFv	pelB/pET22b	1 mM IPTG	25 °C	24 h	BL21(DE3)	LB	pKJE7 DnaK-dnaJ-grpE	65 ug/ml	[109]
	scFv	pelB/pET26b	0.2 mM IPTG	30 °C	4 h	BL21(DE3)	2xYT	DsbA and DsbC	33 mg/L	[170]
	scFv	pelB/pLBAD2				BL21	LB	DsbA	ND	[67]
	scFv	csA11 csB2 /pLBAD2 modified pelB sequence	0.02% arabinose	25 °C	26 h 30 h	BL21A	Lennox Broth		0.65 g/L Fermentation	[98]
**EXTRACELLULAR**	scFv	pelB/pET26 His-tag C terminal	0.1 mM IPTG 0.25% TRITON	25 °C	12 h	BL21(DE3)	M9-glucose medium		2.86 mg/L	[159]
	Fab	phoA/STII pRSFDuet modified	Starvation	20 °C	16 h	BL21(DE3)	LB and PLM medium		up to 10 mg/L	[150]
